# Densities of Bornean orang‐utans (*Pongo pygmaeus morio*) in heavily degraded forest and oil palm plantations in Sabah, Borneo

**DOI:** 10.1002/ajp.23030

**Published:** 2019-07-21

**Authors:** Dave J.I. Seaman, Henry Bernard, Marc Ancrenaz, David Coomes, Thomas Swinfield, David T. Milodowski, Tatyana Humle, Matthew J. Struebig

**Affiliations:** ^1^ Durrell Institute of Con servation and Ecology (DICE), School of Anthropology and Conservation University of Kent Canterbury UK; ^2^ Institute for Tropical Biology and Conservation Universiti Malaysia Sabah Kota Kinabalu Sabah Malaysia; ^3^ HUTAN‐Kinabatangan Orangutan Conservation Programme Sandakan Sabah Malaysia; ^4^ Borneo Futures Bandar Seri Begawan Brunei Darussalam; ^5^ Department of Plant Sciences, Forest Ecology and Conservation Group University of Cambridge Cambridge UK; ^6^ Centre for Conservation Science Royal Society for the Protection of Birds, David Attenborough Building Cambridge UK; ^7^ School of GeoSciences University of Edinburgh Edinburgh UK

**Keywords:** habitat disturbance, human‐modified tropical landscape, LIDAR, oil palm, orang‐utan, Pongo pygmaeus morio

## Abstract

The conversion of forest to agriculture continues to contribute to the loss and fragmentation of remaining orang‐utan habitat. There are still few published estimates of orang‐utan densities in these heavily modified agricultural areas to inform range‐wide population assessments and conservation strategies. In addition, little is known about what landscape features promote orang‐utan habitat use. Using indirect nest count methods, we implemented surveys and estimated population densities of the Northeast Bornean orang‐utan (*Pongo pygmaeus morio*) across the continuous logged forest and forest remnants in a recently salvage‐logged area and oil palm plantations in Sabah, Malaysian Borneo. We then assessed the influence of landscape features and forest structural metrics obtained from LiDAR data on estimates of orang‐utan density. Recent salvage logging appeared to have a little short‐term effect on orang‐utan density (2.35 ind/km ^2^), which remained similar to recovering logged forest nearby (2.32 ind/km ^2^). Orang‐utans were also present in remnant forest patches in oil palm plantations, but at significantly lower numbers (0.82 ind/km ^2^) than nearby logged forest and salvage‐logged areas. Densities were strongly influenced by variation in canopy height but were not associated with other potential covariates. Our findings suggest that orang‐utans currently exist, at least in the short‐term, within human‐modified landscapes, providing that remnant forest patches remain. We urge greater recognition of the role that these degraded habitats can have in supporting orang‐utan populations, and that future range‐wide analyses and conservation strategies better incorporate data from human‐modified landscapes.

## Research Highlights

- Forest remnants within oil palm estates can support substantial orang‐utan numbers, although at reduced density.
- Orang‐utan density is more influenced by forest structure than isolation from large forested areas in human‐modified landscapes of Borneo.

## 1 INTRODUCTION

Agriculture is a leading cause of deforestation globally and, with increasing demands for food and commodities, this trend is likely to continue (Sandker, Finegold, D'annunzio, & Lindquist, 2017). Inevitably, deforestation leads to losses of biodiversity and ecosystem services (Chapin Iii et al., 2000), and nowhere are these losses felt more than in high biodiversity tropical regions (Pimm & Raven, 2000). Southeast Asia has experienced some of the highest deforestation rates in the world (Hansen et al., 2013). Deforestation has been particularly severe in Borneo, Sumatra and Peninsular Malaysia, which, between 2000 and 2010 lost roughly 11% of their lowland forests and 20% of peatswamp forest (Miettinen, Shi, & Liew, 2011). Forests in the region have been cleared for commercial plantations, such as rubber, timber, and fast‐growing trees for the pulp and paper industry, but clearance for oil palm (*Elaeis guineensis*) has been particularly extensive over the last 20 years (Gaveau et al., 2016). Mitigating the negative effects of further forest conversion presents a huge challenge for conservationists, balancing the needs of developing nations whilst protecting biodiversity and the valuable ecosystem services they provide.

Orang‐utans (*Pongo* spp.) are the only non‐human great ape found outside Africa. Although heralded as conservation icons (Meijaard, Wich, Ancrenaz, & Marshall, 2012) and under strict legal protection across all range states, all three species of orang‐utans are Critically Endangered on the IUCN Red List of Threatened Species (IUCN, 2017). Habitat loss, fragmentation, and hunting continue to be leading contributors of population decline (Meijaard et al., 2011; Voigt et al., 2018; Wich et al., 2016), and could have particularly catastrophic consequences in combination with range contractions expected under climate change (Struebig et al., 2015). Currently, the highest densities of orang‐utans are in forests lower than 500 m above sea level (ASL; Voigt et al., 2018). However, these low‐lying areas are often the most suitable for agriculture, leading to high levels of deforestation and forest degradation within the orang‐utan range (Santika et al., 2017). Further forest conversion is expected, and estimates of future orang‐utan habitat loss range from 23,000 km^2^ to as much as 57,000 km^2^ by the 2050s (9–20% reduction; Struebig et al., 2015).

Orang‐utan dietary and behavioral ecology makes these species highly adapted to tropical forests (Marshall et al., 2009). Orang‐utans prefer moving through areas of uniform canopy height, avoid forest gaps (Felton, Engström, Felton, & Knott, 2003), and face energetic costs associated with their arboreal habits (Davies, Ancrenaz, Oram, & Asner, 2017). Although orang‐utans will readily move on the ground through areas of oil palm, most observations in oil palm (nests or signs of feeding) are within 50 m of forest areas (Ancrenaz et al., 2015). The forest canopy buffers against extreme temperature changes and solar radiation (Hardwick et al., 2015) and likely provide important refuge, resources, and nesting opportunities for orang‐utans in heavily modified landscapes. Therefore, three‐dimensional structural features of the canopy are likely to be important determinants of orang‐utan presence.

On Borneo, an estimated 78% of the island's orang‐utan (*pongo pygmaeus*) population is outside of protected areas (Wich et al., 2012). Therefore, the inclusion of human‐modified landscapes within orang‐utan conservation strategies will be vital to ensure the species’ long‐term persistence (Ancrenaz et al., 2015). Recent efforts have yielded valuable information on distributions (Husson et al., 2009; Voigt et al., 2018; Wich et al., 2012), population trends (Santika et al., 2017), responses to future human and climate‐driven land cover changes (Struebig et al., 2015; Wich et al., 2016), as well as the effects of habitat disturbance (Ancrenaz, et al., 2015, 2010; Spehar & Rayadin, 2017). However, there is still a paucity of data on orang‐utan density, demographic response, and dispersal within anthropogenic landscapes, which is vital to inform effective conservation initiatives.

Here we employ orang‐utan nest surveys to determine orang‐utan population densities in the continuous logged forest and forest remnants in a recently salvage‐logged area and oil palm plantations in Sabah. We explore the role of forest structural data and landscape features in predicting orang‐utan density.

## 2 METHODS

### 2.1 Study site

**Figure 1:**
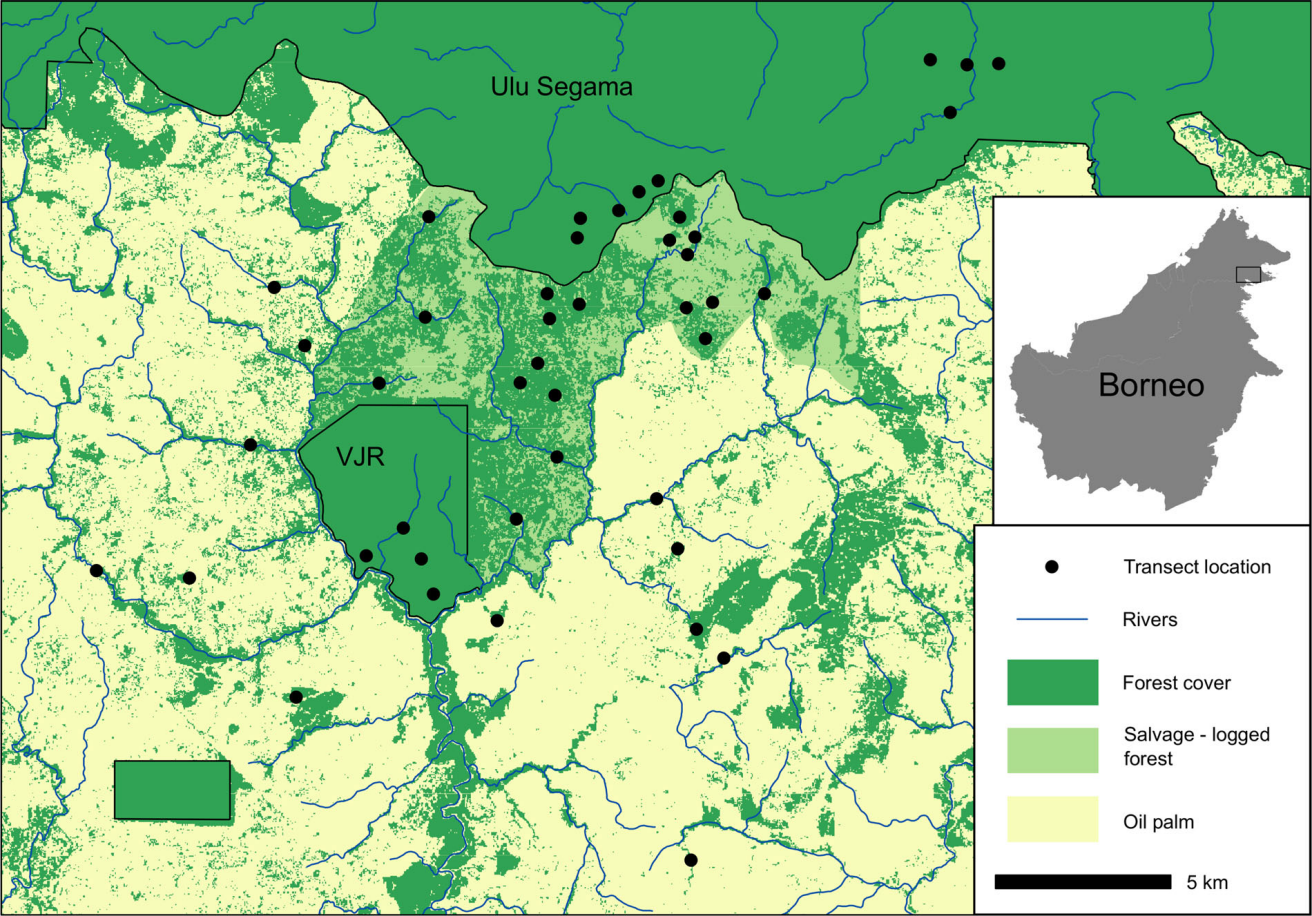
Placement of transects across the study landscape in Sabah, Borneo

Our study was conducted in and around the Stability of Altered Forest Ecosystems project (SAFE: https://www.safeproject.net), including the Kalabakan and Ulu Segama forest reserves and surrounding oil palm estates in the Malaysian state of Sabah, Borneo. The total study area comprises 13,000 ha, of which 7,200 ha is within the SAFE experimental area, which is being converted to oil palm plantation (Ewers et al., 2011; Struebig et al., 2013). Most of the forest has experienced several rounds of logging since 1978, yet still supports substantial primate biodiversity (Bernard et al., 2016). The SAFE area was later salvage‐logged (removal of all remaining commercially valuable trees) between 2013 and 2016, with some areas retained as forest fragments for scientific research (Figure 1). To the north, a block of continuous twice‐logged forest in Ulu Segama connects to >1 million ha of forest habitat, including pristine conservation areas, such as Danum Valley and Maliau Basin. Ulu Segama contains one of the largest unfragmented populations of orang‐utans in Malaysia (2,300 individuals), which is thought to have remained relatively stable since initial surveys in 2002 (Ancrenaz et al., 2010). The wider landscape also contains a substantial block of old growth forest, the Brantian‐Tatulit Virgin Jungle Reserve (VJR), which covers 2,200 ha, although logging encroachment has caused considerable degradation across much of the reserve (Deere et al., 2018). The remainder of the site comprises oil palm estates, which were 8–12 years old at the time of this study. These estates contain remnant forest patches, many of which are within riparian reserves between 15 and 500 m wide (Mitchell et al., 2018).

### 2.2 Transect design

To investigate the effects of habitat modification on orang‐utan abundance, we placed transects and surveyed orang‐utan nests within three distinct habitat types representative of the wider landscape, as well as other parts of the oil palm producing regions of Borneo and Sumatra. These included:

- 13 transects in the logged forest of Ulu Segama Forest Reserve and Brantian‐Tantulit VJR;
- 19 transects in newly isolated remnant forest patches and riparian reserves within the salvage‐logged SAFE experimental area;
- 12 transects in forest remnants (hillside fragments and riparian reserves) within oil palm estates.

Transect length ranged between 0.6 km and 2 km, with an average of 1.6 km across the three habitats. We ensured > 3 km of transect within each habitat, as this is the minimum length needed to produce density estimates in areas of low orang‐utan density (Singleton, 2000). To ensure spatial independence in sampling, transects were placed randomly at least 500 m apart, or were implemented on pre‐existing routes established independently as part of the SAFE project experimental design. Transects in riparian remnants followed the river course to ensure the survey remained within the forest area and avoided oversampling the oil palm matrix. In total, 44 transects were surveyed once, with a combined survey effort of 51.3 km.

### 2.3 Orang‐utan nest surveys

Orang‐utans build nests daily to rest during the day and to sleep in overnight (van Casteren et al., 2012). These nests are complex and have characteristics that make them easily distinguishable from those made by sympatric species, such as sun bear (*Helarctos malayanus*), giant squirrel (*Ratufa affinis*), or raptors (van Casteren et al., 2012). Typically, a minimum of 60–80 nest observations is required to reliably estimate great ape densities using distance‐based methods (Kühl, Maisels, Ancrenaz & Williamson, 2008).

We conducted nest surveys between April and August 2017, using the standing crop methods described by Spehar et al. (2010). Transects were surveyed once by walking at a steady pace, stopping at regular intervals to scan every direction for nests. Upon nest encounter, we measured the perpendicular distance from directly under the nest to the transect line, using a tape measure. We assigned a decay category to each nest, ranging from A to E: where A = new nest, solid structure and leaves still green, B = leaves have started to dry out and discolor, C = nest structure still intact, leaves starting to disappear, D = most leaves gone, nest structure starting to disintegrate, and E = all leaves gone, structure visible but heavily degraded (Spehar et al., 2010).

### 2.4 Parameters in the orang‐utan density model

Conversions of nest density to orang‐utan density requires three parameters: Proportion of nest builders within the population (*p*), nest production rate (*r*), and the nest decay rate (*t*). Because no measures were available for the site, we incorporated parameters from the published literature. We employed a conservative estimate of the proportion of nest producers (*p*) at .85 and used a nest production rate (*r*) value of 1.00, reported from a long‐term study in the Lower Kinabatangan in Sabah (Ancrenaz, Calaque, & Lackman‐Ancrenaz, 2004). As nest decay rate (*t*) shows the highest variation across sites, we calculated orang‐utan density using a rigorously estimated *t* value of 259 days, reported from Gunung Palung (Johnson, Knott, Pamungkas, Pasaribu, & Marshall, 2005). We chose *t* from Gunung Palung as this is from a similar forest type and calculated from a large number of nests over an extended period. However, because changes in environmental conditions, altitude, and rainfall have been reported to influence decay rate (Mathewson, Spehar, Meijaard, Sasmirul, & Marshall, 2008), comparing estimates from across a disturbance gradient using the same decay rate may not always be suitable. As we used parameters obtained from other sites, our results are best interpreted as relative measures of density between habitat types.

### 2.5 Sensitivity analysis

To test the robustness of our density estimates to uncertainties surrounding the three demographic and nest visibility parameters used in the orang‐utan density model, we performed a sensitivity analysis. We reproduced density estimates using high, medium (our original estimate), and low values, for each of the three input parameters *t, r*, and *p*. Parameter combinations resulted in 27 possible iterations, allowing us to examine variation in estimates via histograms. We produced three subsets of estimates, whereby one parameter was fixed at the medium value and the other two varied across all possible combinations of high, medium, and low values, allowing for the effect of each individual parameter on the density estimate to be examined. For *t*, we used the highest (602: Bruford et al., 2010) and lowest values reported for Borneo (202: Ancrenaz et al., 2004), compared with the medium value (259: Johnson et al., 2005). For *r*, we already incorporated the lowest value available in the literature of 1.00, therefore, we used the highest available value (1.16: Johnson et al., 2005) and subtracted the difference between the high and medium values from the medium value, given a low *r* value of 0.84, which would be indicative of high levels of nest reuse. Similarly, for *p*, we already utilized a conservative value of 0.85 and, therefore, we used a high value of 0.88 (van Schaik, Wich, Utami, & Odom, 2005), resulting in a low value of 0.82.

### 2.6 Calculating nest density

We calculated nest encounter rate by dividing the number of nests recorded along each transect by the total survey effort. As there was no significant difference in the distribution of perpendicular distances across the three habitat types (*X* ^*2*^ = 1.080, *df* = 2, *P* = .583), we were able to compare nest encounter rates between habitat types.

We obtained nest density using the formula:

$$D_{nest}\;=N\;/\left(L⁎2w\right)$$

Where *N* is the number of nests observed along each transect, *L* is the length of each transect and *w* is the effective strip width, calculated using Distance software 7.1 (Thomas et al., 2010). Examination of histograms of the data suggested density estimates were slightly spiked at zero, therefore, the data were aggregated into distance classes at 4 m intervals. Similarly, to avoid biases from outliers, we truncated data at 40 m. Six distance models were fitted to the data‐uniform key with either cosine or simple polynomial adjustments, half‐normal key with either cosine or hermite polynomial adjustments and hazard‐rate key with cosine and simple polynomial adjustments. We then assessed model fit using the Chi‐Square goodness of fit test (*X* ^*2*^), and obtained estimates of *w* from the best performing model, using Akaike Information Criterion (AIC) values. As we observed sufficient numbers of nests within each habitat type, we fitted detection functions to pooled data from each habitat type separately.

We then converted nest densities to orang‐utan density using the formula:

$$D_{orang}\;=D_{nest}\;/\left(p⁎r⁎t\right)$$

Where *p* is the proportion of nest builders within the population, *r* is nest production rate and *t* is nest decay rate. To assess possible associations between orang‐utan density and environmental correlates, we calculated orang‐utan densities individually for each transect or fragment and produced estimates of error around the mean density of each habitat type.

### 2.7 Predictors of orang‐utan density

To identify potential predictors of orang‐utan density in the heavily modified landscape, we obtained vegetation structural metrics from airborne LiDAR data, collected by NERC's Airborne Research Facility between September and October 2014 (Jucker et al., 2018). A detailed description of the data collection and processing is available in Jucker et al. (2018). Briefly, ground points from the georeferenced point cloud were classified into ground and non‐ground returns, with a digital elevation model (DEM) produced from the ground data. A normalized canopy height model CHM was produced by subtracting the DEM from the non‐ground returns. The CHM was then used to generate two derived raster products describing the three‐dimensional vegetation structure: (a) a 50 cm resolution pit‐free top of canopy height raster; and (b) a 20 m resolution stack of plant area index (PAI in m^2^ m^−2^; strictly plant area density) rasters, measuring the one‐sided area of leaves and woody tissues per unit surface area, through 1 m deep vertical canopy profile slices. Total PAI was calculated as the sum of the vertical slices and PAI diversity was calculated using the Shannon index across all of the vertical slices (see Table 1).

**Table 1:** Predictor variables for linear models. LiDAR‐based metrics were averaged within a 40 m buffer of each transect

Predictor variables	Description
Local‐level (from LiDAR)	
Canopy height	Mean height of canopy within the buffer.
Canopy height variation	Standard deviation of canopy height. A measure of heterogeneity in the canopy.
No. layers	Number of contiguous layers within the vertical forest column.
Shannon index	Index of diversity in the distribution of material within the vertical column.
Landscape‐level	
Habitat type	The habitat type in which the transect was embedded.
Forest cover	Percentage forest cover within a 150 ha buffer around each transect
Distance	Distance to the nearest continuous logged forest, measured from the midpoint of each transect to the closest border with either Ulu Segama Forest Reserve or the VJR.

In addition to the LiDAR‐based information, we investigated landscape‐level features as possible predictors of orang‐utan densities, because these measures influence densities elsewhere in Borneo. Spehar and Rayadin (2017) found orang‐utan abundance to increase with proximity to natural forest. Therefore, we also included the distance from the nearest large forest area (Ulu Segama or the VJR) and the percentage of forest cover within a 150 ha buffer (typical home range of a female orang‐utan in a heavily disturbed forest; Ancrenaz Unpublished Data) around each transect, as possible predictors. We derived these covariates using layers produced by Hansen et al. (2013) to reflect forest cover at the time of our surveys (See Table 1). For pairwise comparison of predictor variables among habitat types see supporting information (Figure S1).

### 2.8 Statistical analysis

Both nest encounter rate and orangutan density estimates were normally distributed (Shapiro‐Wilk test, W = 0.958, *P = *.304 and W = 0.969, *P* = .553, respectively) and had homogeneous variance between habitat types (Bartlett's test K = 2.434, *df* = 2, *P* = .296 and K = 1.832, *df* = 2, *P* = .400, respectively). We, therefore, employed a One‐Way ANOVA to assess differences in nest encounter rate and orang‐utan density between habitat types. To assess relationships between nest encounter rate and orang‐utan density, relative to several landscape and forest structural predictor variables, we used multiple linear regression models (LM). We applied LMs with a Gaussian error structure and identity link function to the data. LMs were specified with an effects parameterisation, designating logged forest as the fixed intercept and reference habitat class from which to assess deviations in the response variable. Using methods delineated by Grueber, Nakagawa, Laws, and Jamieson (2011), we fitted a global model to the data that included all predictor variables. Using the R package *arm* (Gelman & Su, 2018), we standardized variables to have a mean of 0 and a standard deviation of 0.5, to enable the direct comparison of the effect size of parameter estimates derived from model averages. The dredge function was then applied to the global model using the *MuMIn* package (Barton, 2009), which produces a set of all possible model outcomes, including an intercept‐only model. Predictor variables were examined for collinearity using the Pearson product‐moment correlation coefficient (*r*) and generalized variance inflation factors (GVIF), with variables considered highly collinear if *r ≥ *0.7 or GVIF ≥ 5 (Zuur, Ieno, & Elphick, 2010). We observed a high degree of collinearity among variables and as a result, we coded models to exclude highly collinear variables from appearing in the same model.

We ranked models based on corrected AIC scores. Across all models, parameter estimates were averaged and parameters weighed on the basis of the proportion of models in which each was included (Grueber et al., 2011). We inspected residual diagnostics to determine the influences of curvature and heteroscedasticity, considered indicative of poor model fit. Model validation identified a single outlier with high leverage (Cook's Distance > 1). Because subsequent removal and reanalysis found no significant effect on the parameter estimates, we present findings for models including the outlying data point. All analysis was performed using R version 3.4.2 statistical software (R Core Team, 2017). The data will be available from the NERC Environmental Information Data Center following an embargo period (accessible from 18th March 2021, https://doi.org/10.5281/zenodo.3237506)

### 2.9 Ethical statement

The study was approved by the University of Kent's Animal Welfare Ethics Review Board and fully complied the American Society of Primatologists Principles for the Ethical Treatment of Non‐Human Primates. Field research was authorized by Sabah Biodiversity Council under access license No. JKM/MBS.1000–2/2 JLD.4(104).

## 3 RESULTS

We observed 678 nests along the 44 transects. After transects outside the LiDAR extent were excluded and the data were truncated, 594 nests on 35 transects remained for analyses.

### 3.1 Orang‐utan density

**Figure 2:**
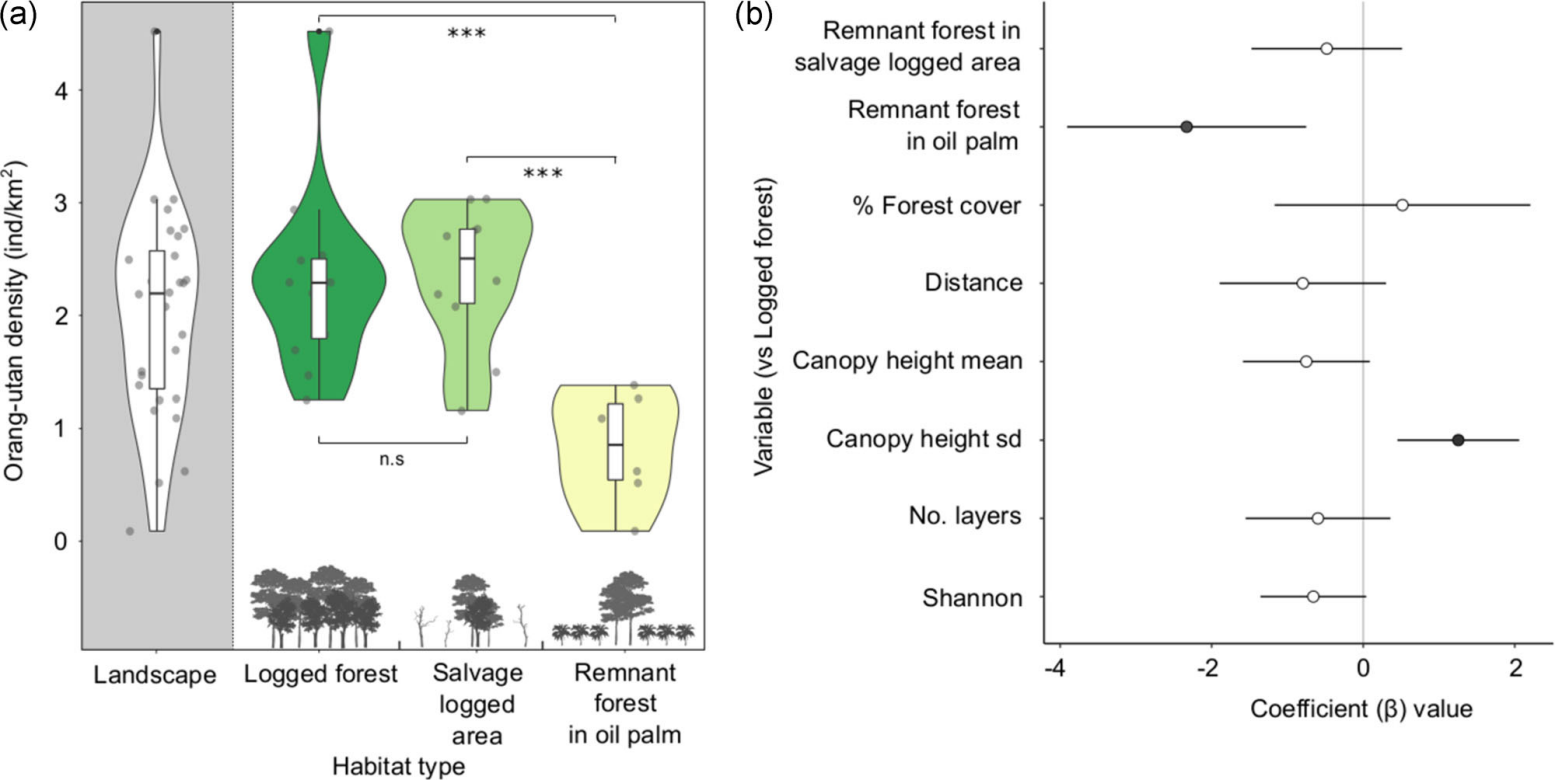
(a) Violin plots of orang‐utan density (individuals/km2), for the overall landscape and between habitat types. A significant difference of *p*< 0.001 between habitat types is denoted by *** and no significance by n.s. Data points are jittered for visualization. (b) Coefficient plot (β) from an averaged model of orang‐utan population density, showing 95% confidence intervals

Over the whole landscape, we encountered an average of 13.31 nests/km, and generated an estimate of 2.01 orang‐utans per km^2^ (Table 2). However, both nest encounter rate and resulting density estimates varied considerably across the landscape (nest encounter rate, 0.56‐30.83 nests/km; density, 0.09‐4.52 ind/km^2^), with overall significant differences among habitats (ANOVA: nests, *F*_2, 12_ = 15.49, *P* = < .001; density *F*_2, 24_ = 15.37, *P* = < .001). Density estimates were similar between logged forest and forest remnants in the salvage‐logged area (mean 2.32 and 2.35, respectively; Tukey post hoc test, *P *= .601), but were significantly lower in the forest remnants in the oil palm (mean 0.82, *P = *< .001; Figure 2).

**Table 2:** Summary of nest‐count survey data

Habitat Type	Site ID	No. of nests	Transect length (km)	Effective strip width^a^ (m)	Nest encounter rate (nests/km)	Orangutan density (Ind/km^2^)
Continuous logged forest						
	LF1	31	1.8	15.5	17.2	2.5
	LF2	23	2	15.5	11.5	1.7
	LF3	25	2	15.5	12.5	1.8
	LFR	15	1	15.5	15.0	2.2
	LFE1	17	2	15.5	8.5	1.3
	LFE2	24	1.5	15.5	15.7	2.3
	LFE3	24	1.2	15.5	20.0	2.9
	LFE4	17	1	15.5	17.0	2.5
	LFER	25	1.6	15.5	15.6	2.3
	VJR_R	25	1.6	15.5	15.6	2.3
	VJR_1	37	1.2	15.5	30.8	4.5
	VJR_2	10	1	15.5	10.0	1.5
Salvage‐logged forest						
	RR0	30	1.6	14.3	19.1	3.0
	RR5	26	1.5	14.3	17.3	2.8
	RR15	28	1.6	14.3	17.5	2.8
	RR30	29	1.7	14.3	17.1	2.7
	RR60	11	1.5	14.3	7.3	1.2
	RR120	21	1.6	14.3	13.1	2.1
	Block_B	28	1.9	14.3	14.6	2.3
	Block_C	29	2.1	14.3	13.8	2.2
	Block_D	24	2.4	14.3	9.5	1.5
	Block_E	43	2.3	14.3	19.1	3.0
Forest remnants in oil palm plantations						
	OP02	13	1.6	14.7	8.1	1.3
	OP03	9	1.3	14.7	7.0	1.1
	OP07	1	1.8	14.7	0.6	0.1
	OP12	6	1.8	14.7	3.4	0.5
	OP14	16	1.8	14.7	8.9	1.4
	OP16	7	1.8	14.7	4.0	0.6

^a^ Effective strip width was calculated in Distance 1.7 software (Thomas et al., 2010).

### 3.2 Landscape determinants of orang‐utan density

Our information‐theoretic statistical approach yielded 48 possible models (Tables S1,S2) from which we produced full model‐averaged estimates penalized for parameter redundancy. For habitat type, these models confirmed that nest encounter rate and orang‐utan densities were lower in remnant forest patches in oil palm (Coefficient β = −16.44, 95%CI = −26.48, − 6.39 and β = −2.33, 95%CI = −3.91, −0.75, respectively, Figure 2). Variation in canopy height was also positively associated with nest encounters and densities (β = 7.76, 95%CI = 2.62, 12.90 and β = 1.25, 95%CI = 0.45, 2.06, respectively). The 95% confidence intervals of all other variables crossed zero, indicating that they had little effect on orang‐utan abundance.

### 3.3 Sensitivity analysis

**Figure 3:**
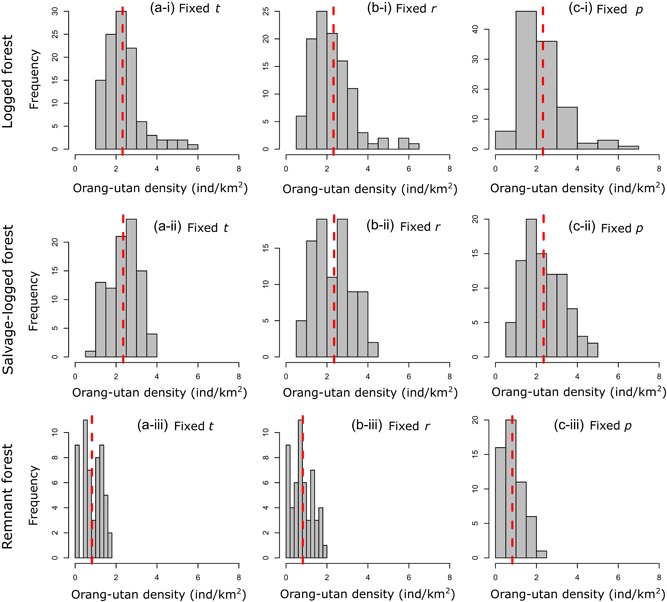
Sensitivity analyses to demonstrate the effect of changing fixed parameters (nest decay rate *t*, nest production rate *r* and proportion of nest builders *p*), on orang‐utan density estimates. The density reported in the main text is labeled by a dashed line in each plot. Plots a, d, and g show results where t is fixed, b, e, and h where r is fixed and c, f, and i where p is fixed, across all three habitat types. For t we used the vales: high 602 (Bruford et al., 2010), medium 259 (Johnson et al., 2005), and low 202 (Ancrenaz et al., 2004). For r: high 1.16 (Johnson et al., 2005), medium 1 (Ancrenaz et al., 2004), and low r value of 0.84. For *p*: high .88 (Van Schail at el., 2005), medium .85 (Ancrenaz et al., 2004), and low value of .82

There was a large range of possible density values, with several estimates substantially higher than our original estimate (Figure 3). For the logged forest, when *t* was fixed, density estimates ranged from 1.03 to 5.65. However, this range increased to between 0.51 and 6.09 when *r* was fixed and *t* and *p* were varied. We observed the largest variation in density estimates when *p* was fixed and both *t* and *r* varied, with estimates increasing to between 0.46 and 6.91. We observed a similar pattern across all habitat types (Table S3).

## 4 DISCUSSION

We produced orang‐utan density estimates across a mosaic landscape in Malaysian Borneo and found orang‐utans were present in all forest habitats, although on average orang‐utan density was ≥65% lower in remnant forest patches in oil palm. The average density across our landscape of 2.01 ind/km^2^, is within the range of estimates produced by Ancrenaz et al. (2010) within the same area from aerial surveys (2.1‐0.7 ind/km^2^).

Recent salvage logging (2–5 years previous to this study) appeared to have little effect on orang‐utan density within remnant forest patches (2.35 ind/km^2^), which was similar in this habitat to neighboring logged forest (Ulu Segama 2.17 ind/km^2^ and the VJR 2.76 ind/km^2^). This result is contrary to previous research that found densities across the orang‐utan range to be higher in areas surrounding recently logged forest (Husson et al., 2009), perhaps because insufficient time had passed to capture the demographic response. At our study site, forest structural metrics revealed that remnant forest patches in the salvage‐logged area are structurally more similar to remnant forest in oil palm, than to areas of logged forest. Although being structurally similar, the SAFE experimental area has been disturbed relatively recently 2–5 years before the study) compared to the remnant forest patches in the oil palm estates 8‐12 years). Orang‐utans have the longest interbirth period of any mammal (ca. 9 years) and an extended period of adolescence before first birth (Knott, Emery Thompson, & Wich, 2009). This long life history may result in a large time lag before demographic responses to disturbance are truly observed, meaning that there may have been insufficient time for the full effects of the disturbance on orang‐utan populations to manifest in the SAFE experimental area.

The salvage‐logged area at SAFE is due to be converted to oil palm. On the basis of the density of nests, the area still appears to support a relatively large number of orang‐utans. During the conversion process, any remaining vegetation will be felled and cleared before terracing and the planting of oil palm commences, forcing most wildlife, including orang‐utans, to move to the neighboring forest areas or become isolated in remnant forest patches. In our study area, the nearby Ulu Segama and the VJR already support high densities of orang‐utans, and immigration of orang‐utans from surrounding areas would increase competition for resources. In addition, the VJR will be fragmented when the oil palm is planted. Unless a wildlife corridor is maintained to link the VJR to Ulu Segama, orang‐utans in this forest will be isolated and overcrowded unless they are able to disperse across several kilometers of oil palm plantation. A recent integrative trend analysis found orang‐utan survivorship was lowest in areas of fragmented forest or near to areas of recent forest conversion to agriculture (Santika et al., 2017). With increasing areas of orang‐utan habitat likely to be converted to oil palm, practical matters need to be considered to ensure resident animals can disperse successfully.

Both indices of orang‐utan abundance in remnant forest patches within oil palm estates were lower than those in the logged forest and remnant forest in the salvage‐logged area. As expected, conversion to oil palm has a negative effect on local orang‐utan populations. Despite these negative effects, we encountered nests on all transects within remnant forest patches and riparian reserves in oil palm estates. We also directly observed three adult females with dependent young within several riparian reserves, suggesting reproductive orang‐utans use these areas. Additionally, as nests were observed at large distances (≥6 km) from the nearest large forest area, it is likely at least some of these individuals are resident within the estates. Equally, because densities were similar in salvage‐logged and logged forest, it is doubtful that orang‐utans within oil palm estates are those displaced during the salvage logging process. The above observations suggest that the oil palm plantation still hosts an orang‐utan population, albeit at a lower density than in the logged forest.

The linear models revealed certain nuances in the data that may be important in explaining orang‐utan persistence within oil palm estates. Although we expected distance from logged forests to have a negative effect on orang‐utan presence, we found no evidence in our survey that distance from this forest affects orang‐utan density. Davies at el. (2017) found the number of contiguous layers in the canopy did not determine orang‐utan movement through disturbed forests in the Kinabatangan region. Similarly, we found little evidence that vertical layering had an effect on orang‐utan densities across our study landscape. However, contrary to Davies at el. (2017), we found that large variation in canopy height was positively associated with orang‐utan density. Across our study site, the most heavily degraded areas tended to be dominated by pioneer species, such as *Macaranga* spp (Struebig et al., 2013), giving the canopy a highly uniform structure. Orang‐utans also appear averse to nesting within *Macaranga* spp., and therefore these areas may be ecologically unsuitable to support orang‐utans (Ancrenaz et al., 2004). Variation in canopy height is strongly associated with successional status (Deere et al. (2018) Unpublished Data) and thus indicates greater environmental heterogeneity and breadth of resources. Further research is needed to quantify resource availability in remnant forest patches under various levels of degradation and gain an improved understanding of the long‐term carrying capacity of agricultural landscapes. However, at least in our study site, it appears orang‐utans have been able to persist in oil palm estates for several years.

Our sensitivity analysis revealed two important points. First, across all possible iterations of parameter values, the upper limits of our density estimates for remnant forest sites in oil palm were lower than half the upper limits for the logged forest, and density estimates were on average close to a third that of logged forest. These results provide strong evidence that, despite using parameters acquired from other sites in our density calculations, oil palm estates support <50% of the orang‐utan density of the logged forest. Second, our sensitivity analysis corroborates previous research, that density estimates are highly sensitive to changes in nest decay rate (Marshall & Meijaard, 2009). However, our analysis also revealed that nest production rate could have a large influence on density estimates. High levels of disturbance may limit nesting opportunities or alter the abundance of tree species orang‐utans preferentially use for nesting and increase nest reuse (Ancrenaz et al., 2004). If unaccounted for, high levels of nest reuse may potentially lead to an underestimation of orang‐utan density. Therefore, to improve future density estimates in highly modified landscapes, further research is needed to assess orang‐utan nesting behavior within remnant forest patches in oil palm.

Previous research on orang‐utan behavioral ecology in modified landscapes suggests young subordinate males are dispersing from optimal habitat from where they have been displaced by dominant flanged males (Ancrenaz et al., 2015). However, the three orang‐utans we observed directly during our surveys of remnant forest in oil palm were all adult females with dependent offspring. Spehar and Rayadin (2017) also recorded adult females with dependent offspring in timber plantations in East Kalimantan. Orang‐utans exhibit female philopatry and are less likely to disperse over large distances than males (van Noordwijk et al., 2012). Female range fidelity may, therefore, explain the number of females we encountered. Equally, this may indicate female orang‐utans are becoming effectively stranded in heavily degraded landscapes. In any case, our results suggest remnant forest patches in modified landscapes are likely to hold a significant number of reproductive females, which are important to the population and largely overlooked within conservation strategies. Further research is needed to fully understand how these areas affect reproduction and survival rates and the role they play in connecting meta‐populations.

Integrating modified landscapes into orang‐utan conservation strategies poses a significant challenge. Leaving 1,000 ha of land unconverted can entail annual losses to oil palm producers of over US$0.5 million (Nantha & Tisdell, 2009). Despite these potential losses, the oil palm industry is increasingly moving towards business models based on corporate environmental and social responsibility (Morgans et al., 2018). As a result, certification schemes, such as the RSPO, have considerable potential to help conserve orang‐utans within oil palm estates (Nantha & Tisdell, 2009). Across Indonesia, RSPO certification has reduced deforestation by 33% on land managed by certified companies (Carlson et al., 2018). Currently, however, there may be greater numbers of orang‐utans within non‐RSPO certified estates than in certified estates (Morgans et al., 2018). Therefore, increasing the uptake of RSPO certification among oil palm producers will likely reduce deforestation further and aid orang‐utan conservation.

If orang‐utan populations are maintained in human‐modified landscapes, individuals face additional risks which conservationists and policymakers should consider. An increase in orang‐utan proximity to humans is likely to result in a greater risk of zoonotic disease transmission (Russon, 2009). Persecution of orang‐utans is common throughout their range in Borneo (Meijaard et al., 2011). Without adequate law enforcement to eradicate persecution, simply retaining forest fragments is likely to be insufficient to allow orang‐utans to persist in modified landscapes.

The ability of orang‐utans to use modified landscapes is, to some degree, likely to be species‐specific. Bornean orang‐utans display higher dietary flexibility than their Sumatran counterparts (Russon, 2009). Therefore, their ability to cope with reduced food availability is likely to be greater. Furthermore, our study was conducted with the Northeast Bornean orang‐utan (*P. p. morio*), which may be particularly adapted to persist on tough, fall‐back foods, as northeast Borneo is subject to more severe droughts and resource fluctuation as a result of the El Nino Southern Oscillation (Taylor, 2006).

## 5 CONCLUSION

Despite pledges by the Indonesian and Malaysian government to stabilize orang‐utan populations, they have continued to decline by 25% over the past 10 years (Santika et al., 2017). Our results show forest conversion to oil palm negatively affects orang‐utan populations, leading to reduced densities. Nevertheless, we found orang‐utans still persist in remnant forest patches within oil palm estates. The presence of orang‐utans within oil palm estates demonstrates that these great apes may have greater ecological resilience to disturbance than previously assumed. Although forest patches alone cannot maintain viable populations, if managed appropriately, they may act as important corridors or stepping‐stones, connecting isolated populations, and facilitate migration in response to climate change. As orang‐utan habitats are the most suitable areas for oil palm production in Borneo and Sumatra, these modified landscapes should be taken more seriously in orang‐utan conservation and monitoring efforts.

## Supporting information

Supplementary information

Supplementary information

Supplementary information
