# Supplementary material for: Densities of Bornean orang‐utans (Pongo pygmaeus morio) in heavily degraded forest and oil palm plantations in Sabah, Borneo
Source: Am J Primatol. 2019 Jul 21;81(8):e23030. doi: 10.1002/ajp.23030 (PMC6771663; doi:10.1002/ajp.23030)
Supplement: Supplementary file 1 — Supplementary information [file AJP-81-na-s001.docx]

**Densities of Bornean orang-utans (*Pongo pygmaeus morio*) in heavily degraded forest and oil palm plantations in Sabah, Borneo**

Dave J. I. Seaman, Henry Bernard, Marc Ancrenaz, David Coomes, Thomas Swinfield, David T. Milodowski, Tatyana Humle, Matthew J. Struebig

**Comparison of predictor variables among sites**


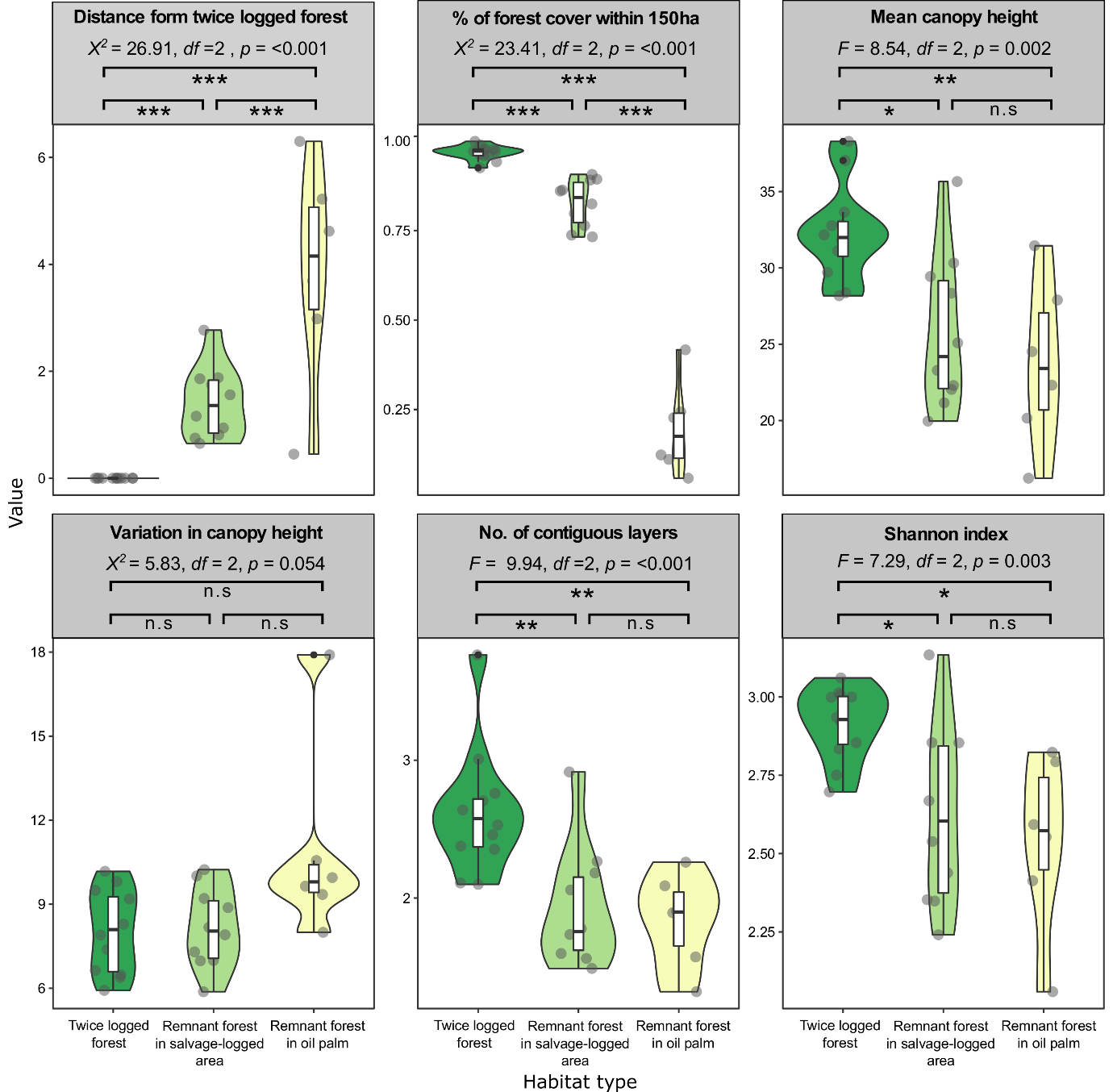


**Figure S1**. Violin plots showing pairwise comparison of each predictor variable among habitat types, using One way ANOVA (*F*) with post hoc Tukey test or Kruskal–Wallis tests (*X^2^*) with post hoc Wilcoxon signed-rank tests. A significance of <0.05 is denoted by *, <0.01 by **, <0.001 by *** and no significance by n.s. Results suggest that remnant forest patches in both the salvage-logged area and oil palm are structurally more similar to each other than to the continuous logged forest.
