# Supplementary material for: Densities of Bornean orang‐utans (Pongo pygmaeus morio) in heavily degraded forest and oil palm plantations in Sabah, Borneo
Source: Am J Primatol. 2019 Jul 21;81(8):e23030. doi: 10.1002/ajp.23030 (PMC6771663; doi:10.1002/ajp.23030)
Supplement: Supplementary file 2 — Supplementary information [file AJP-81-na-s002.docx]

**Densities of Bornean orang-utans (*Pongo pygmaeus morio*) in heavily degraded forest and oil palm plantations in Sabah, Borneo**

Dave J. I. Seaman, Henry Bernard, Marc Ancrenaz, David Coomes, Thomas Swinfield, David T. Milodowski, Tatyana Humle, Matthew J. Struebig

**Multiple linear models**

**Table S1.** Candidate models used to characterize nest encounter rate.

| Model | k† | logLik‡ | AICc§ | ΔAICc¶ | wt¥ |
| --- | --- | --- | --- | --- | --- |
| Encounter rate ~ |  |  |  |  |  |
| Habitat type + Canopy sd + Distance + Shannon | 7 | 140.52 | 295.04 | 0.00 | 0.22 |
| Habitat type + Canopy mean + Canopy sd + Distance | 7 | 140.62 | 295.23 | 0.19 | 0.20 |
| Habitat type + Canopy sd + Shannon | 6 | 141.95 | 295.90 | 0.85 | 0.14 |
| Habitat type + Canopy mean + Canopy sd | 6 | 142.32 | 296.64 | 1.60 | 0.10 |
| Habitat type + Canopy sd + Distance + No layers | 7 | 141.76 | 297.51 | 2.47 | 0.06 |
| Habitat type + Canopy sd + Forest Cover + Shannon | 7 | 141.81 | 297.63 | 2.58 | 0.06 |
| Habitat type + Canopy sd + Distance | 6 | 143.05 | 298.10 | 3.05 | 0.05 |
| Habitat type + Canopy mean + Canopy sd + Forest Cover | 7 | 142.05 | 298.11 | 3.06 | 0.05 |
| Habitat type + Canopy sd | 5 | 144.24 | 298.49 | 3.45 | 0.04 |
| Habitat type + Canopy sd + No layers | 6 | 143.32 | 298.64 | 3.60 | 0.04 |
| Habitat type + Canopy sd + Forest Cover | 6 | 143.96 | 299.93 | 4.88 | 0.02 |
| Habitat type + Canopy sd + Forest Cover + No layers | 7 | 143.00 | 299.99 | 4.95 | 0.02 |
| Canopy sd + Forest Cover + Shannon | 5 | 146.97 | 303.93 | 8.89 | 0.00 |
| Canopy sd + Forest Cover + No layers | 5 | 147.08 | 304.17 | 9.12 | 0.00 |
| Canopy mean + Canopy sd + Forest Cover | 5 | 147.23 | 304.45 | 9.41 | 0.00 |
| Canopy sd + Forest Cover | 4 | 148.70 | 305.39 | 10.35 | 0.00 |
| Habitat type | 4 | 148.93 | 305.86 | 10.82 | 0.00 |
| Habitat type + Shannon | 5 | 147.94 | 305.87 | 10.83 | 0.00 |
| Forest Cover + Shannon | 4 | 149.25 | 306.50 | 11.46 | 0.00 |
| Canopy sd + Distance | 4 | 149.30 | 306.61 | 11.56 | 0.00 |
| Canopy sd + Distance + Shannon | 5 | 148.42 | 306.84 | 11.80 | 0.00 |
| Forest Cover | 3 | 150.48 | 306.96 | 11.92 | 0.00 |
| Habitat type + Forest Cover | 5 | 148.48 | 306.96 | 11.92 | 0.00 |
| Habitat type + Forest Cover + Shannon | 6 | 147.61 | 307.22 | 12.17 | 0.00 |
| Habitat type + Distance + Shannon | 6 | 147.81 | 307.63 | 12.59 | 0.00 |
| Canopy sd + Distance + No layers | 5 | 148.83 | 307.65 | 12.61 | 0.00 |
| Habitat type + Distance | 5 | 148.85 | 307.71 | 12.67 | 0.00 |
| Canopy mean + Canopy sd + Distance | 5 | 148.87 | 307.73 | 12.69 | 0.00 |
| Forest Cover + No layers | 4 | 149.89 | 307.77 | 12.73 | 0.00 |
| Habitat type + Canopy mean | 5 | 148.91 | 307.81 | 12.77 | 0.00 |
| Habitat type + No layers | 5 | 148.91 | 307.83 | 12.79 | 0.00 |
| Canopy mean + Forest Cover | 4 | 150.15 | 308.30 | 13.26 | 0.00 |
| Habitat type + Canopy mean + Forest Cover | 6 | 148.46 | 308.91 | 13.87 | 0.00 |
| Habitat type + Forest Cover + No layers | 6 | 148.46 | 308.91 | 13.87 | 0.00 |
| Habitat type + Canopy mean + Distance | 6 | 148.81 | 309.63 | 14.59 | 0.00 |
| Habitat type + Distance + No layers | 6 | 148.83 | 309.67 | 14.62 | 0.00 |
| Distance | 3 | 153.02 | 312.04 | 17.00 | 0.00 |
| Distance + Shannon | 4 | 152.80 | 313.61 | 18.57 | 0.00 |
| Canopy mean + Distance | 4 | 152.96 | 313.92 | 18.88 | 0.00 |
| Distance + No layers | 4 | 153.01 | 314.03 | 18.98 | 0.00 |
| Null | 2 | 156.41 | 316.82 | 21.78 | 0.00 |
| Canopy mean | 3 | 155.58 | 317.16 | 22.11 | 0.00 |
| No layers | 3 | 155.68 | 317.37 | 22.33 | 0.00 |
| Shannon | 3 | 156.31 | 318.63 | 23.58 | 0.00 |
| Canopy sd | 3 | 156.41 | 318.82 | 23.78 | 0.00 |
| Canopy mean + Canopy sd | 4 | 155.58 | 319.16 | 24.11 | 0.00 |
| Canopy sd + No layers | 4 | 155.68 | 319.36 | 24.32 | 0.00 |
| Canopy sd + Shannon | 4 | 156.31 | 320.62 | 25.58 | 0.00 |

† K indicates the number of parameters used by the model. ‡ The log-likelihood value, an alternative measure of model performance and denotes the plausibility of the model. § Akaike’s Information Criterion AICc. ¶ Delta AICc score, the difference between the AICc score of each model against the best preforming model. ¥ Akaike weight.

**Table S2.** Candidate models used to characterize orang-utan density.

| Model | *k*^†^ | logLik^‡^ | AICc^§^ | ΔAICc^¶^ | wt^¥^ |
| --- | --- | --- | --- | --- | --- |
| Oran-utan density ~ |  |  |  |  |  |
| Habitat type + Canopy sd + Distance + Shannon | 7 | -16.45 | 46.90 | 0.00 | 0.22 |
| Habitat type + Canopy mean + Canopy sd + Distance | 7 | -16.54 | 47.08 | 0.18 | 0.20 |
| Habitat type + Canopy sd + Shannon | 6 | -17.87 | 47.74 | 0.83 | 0.14 |
| Habitat type + Canopy mean + Canopy sd | 6 | -18.24 | 48.47 | 1.57 | 0.10 |
| Habitat type + Canopy sd + Distance + No. layers | 7 | -17.67 | 49.35 | 2.44 | 0.06 |
| Habitat type + Canopy sd + Forest cover + Shannon | 7 | -17.73 | 49.47 | 2.56 | 0.06 |
| Habitat type + Canopy mean + Canopy sd + Forest cover | 7 | -17.97 | 49.93 | 3.03 | 0.05 |
| Habitat type + Canopy sd + Distance | 6 | -18.97 | 49.94 | 3.03 | 0.05 |
| Habitat type + Canopy sd | 5 | -20.16 | 50.31 | 3.41 | 0.04 |
| Habitat type + Canopy sd + No. layers | 6 | -19.23 | 50.46 | 3.56 | 0.04 |
| Habitat type + Canopy sd + Forest cover | 6 | -19.87 | 51.75 | 4.85 | 0.02 |
| Habitat type + Canopy sd + Forest cover + No. layers | 7 | -18.90 | 51.81 | 4.91 | 0.02 |
| Canopy sd + Forest cover + Shannon | 5 | -22.88 | 55.77 | 8.86 | 0.00 |
| Canopy sd + Forest cover + No. layers | 5 | -22.99 | 55.98 | 9.08 | 0.00 |
| Canopy mean + Forest cover | 5 | -23.14 | 56.28 | 9.38 | 0.00 |
| Canopy sd + Forest cover | 4 | -24.61 | 57.23 | 10.32 | 0.00 |
| Habitat type | 4 | -24.85 | 57.71 | 10.80 | 0.00 |
| Habitat type + Shannon | 5 | -23.87 | 57.73 | 10.83 | 0.00 |
| Forest cover + Shannon | 4 | -25.17 | 58.35 | 11.45 | 0.00 |
| Canopy sd + Distance | 4 | -25.23 | 58.46 | 11.55 | 0.00 |
| Canopy sd + Distance + Shannon | 5 | -24.35 | 58.70 | 11.79 | 0.00 |
| Forest cover | 3 | -26.40 | 58.80 | 11.90 | 0.00 |
| Habitat type + Forest cover | 5 | -24.41 | 58.81 | 11.91 | 0.00 |
| Habitat type + Forest cover + Shannon | 6 | -23.54 | 59.07 | 12.17 | 0.00 |
| Habitat type + Distance + Shannon | 6 | -23.74 | 59.48 | 12.58 | 0.00 |
| Canopy sd + Distance + No. layers | 5 | -24.75 | 59.50 | 12.60 | 0.00 |
| Habitat type + Distance | 5 | -24.78 | 59.55 | 12.65 | 0.00 |
| Canopy mean + Canopy sd + Distance | 5 | -24.79 | 59.59 | 12.68 | 0.00 |
| Forest cover + No. layers | 4 | -25.81 | 59.61 | 12.71 | 0.00 |
| Habitat type + Canopy mean | 5 | -24.83 | 59.66 | 12.76 | 0.00 |
| Habitat type | 5 | -24.84 | 59.68 | 12.77 | 0.00 |
| Canopy mean + Forest cover | 4 | -26.07 | 60.15 | 13.24 | 0.00 |
| Habitat type + Canopy mean + Forest cover | 6 | -24.38 | 60.76 | 13.85 | 0.00 |
| Habitat type + Forest cover | 6 | -24.38 | 60.76 | 13.85 | 0.00 |
| Habitat type + Canopy mean + Distance | 6 | -24.74 | 61.47 | 14.57 | 0.00 |
| Habitat type + Distance | 6 | -24.76 | 61.51 | 14.61 | 0.00 |
| Distance | 3 | -28.95 | 63.89 | 16.99 | 0.00 |
| Distance + Shannon | 4 | -28.73 | 65.46 | 18.56 | 0.00 |
| Canopy mean + Distance | 4 | -28.88 | 65.77 | 18.86 | 0.00 |
| Distance + No. layers | 4 | -28.94 | 65.88 | 18.97 | 0.00 |
| Null | 2 | -32.32 | 68.64 | 21.74 | 0.00 |
| Canopy mean | 3 | -31.49 | 68.98 | 22.08 | 0.00 |
| No. layers | 3 | -31.60 | 69.20 | 22.29 | 0.00 |
| Shannon | 3 | -32.22 | 70.45 | 23.54 | 0.00 |
| Canopy sd | 3 | -32.32 | 70.64 | 23.74 | 0.00 |
| Canopy mean + Canopy sd | 4 | -31.49 | 70.98 | 24.08 | 0.00 |
| Canopy sd + No. layers | 4 | -31.59 | 71.19 | 24.28 | 0.00 |
| Canopy sd + Shannon | 4 | -32.22 | 72.44 | 25.53 | 0.00 |

† *k* indicates the number of parameters used by the model. ‡ The log-likelihood value, an alternative measure of model performance and denotes the plausibility of the model. § Akaike’s Information Criterion AICc. ¶ Delta AICc score, the difference between the AICc score of each model against the best preforming model. ¥ Akaike weight.
