# Supplementary material for: Densities of Bornean orang‐utans (Pongo pygmaeus morio) in heavily degraded forest and oil palm plantations in Sabah, Borneo
Source: Am J Primatol. 2019 Jul 21;81(8):e23030. doi: 10.1002/ajp.23030 (PMC6771663; doi:10.1002/ajp.23030)
Supplement: Supplementary file 3 — Supplementary information [file AJP-81-na-s003.docx]

**Densities of Bornean orang-utans (*Pongo pygmaeus morio*) in heavily degraded forest and oil palm plantations in Sabah, Borneo**

Dave J. I. Seaman, Henry Bernard, Marc Ancrenaz, David Coomes, Thomas Swinfield, David T. Milodowski, Tatyana Humle, Matthew J. Struebig

**Sensitivity analysis**

|  |  | | Orang-utan density | | |  | |
| --- | --- | --- | --- | --- | --- | --- | --- |
| Habitat type | | Fixed  parameter | Mean | Max | Min | | SD |
| Logged forest | |  |  |  |  | |  |
|  | | Reported^†^ | 2.32 | 4.52 | 1.25 | | 0.81 |
|  | | *t* fixed | 2.36 | 5.58 | 1.04 | | 0.89 |
|  | | *r* fixed | 2.10 | 6.01 | 0.52 | | 1.14 |
|  | | *p* fixed | 2.13 | 6.91 | 0.46 | | 1.20 |
| Salvage-logged forest | | |  |  |  | |  |
|  | | Reported^†^ | 2.35 | 3.03 | 1.16 | | 0.60 |
|  | | *t* fixed | 2.39 | 3.74 | 0.97 | | 0.70 |
|  | | *r* fixed | 2.13 | 4.02 | 0.48 | | 1.02 |
|  | | *p* fixed | 2.16 | 4.62 | 0.43 | | 1.08 |
| Remnant forest in oil palm | | |  |  |  | |  |
|  | | Reported^†^ | 0.82 | 1.38 | 0.09 | | 0.45 |
|  | | *t* fixed | 0.84 | 1.70 | 0.07 | | 0.48 |
|  | | *r* fixed | 0.75 | 1.83 | 0.04 | | 0.53 |
|  | | *p* fixed | 0.76 | 2.10 | 0.03 | | 0.55 |

**Table S3.** Results of sensitivity analysis.

^†^Figures reported in this study
